# Absence of Polyphenol Oxidase in *Cynomorium coccineum,* a Widespread Holoparasitic Plant

**DOI:** 10.3390/plants9080964

**Published:** 2020-07-30

**Authors:** Alessandra Padiglia, Paolo Zucca, Faustina B. Cannea, Andrea Diana, Cristina Maxia, Daniela Murtas, Antonio Rescigno

**Affiliations:** 1 Dipartimento di Scienze della vita e dell’ambiente (Disva), Cittadella Universitaria di Monserrato, 09042 Monserrato, Italy; padiglia@unica.it (A.P.); faustinabarbara@tiscali.it (F.B.C.); 2 Dipartimento di Scienze biomediche (DiSB), Cittadella Universitaria di Monserrato, 09042 Monserrato, Italy; pzucca@unica.it (P.Z.); diana@unica.it (A.D.); cmaxia@unica.it (C.M.); murtas@unica.it (D.M.)

**Keywords:** antioxidants, Cynomoriaceae, tyrosinase, parasitic plants, phenolics, drought

## Abstract

Polyphenol oxidase (PPO, E.C. 1.14.18.1) is a nearly ubiquitous enzyme that is widely distributed among organisms. Despite its widespread distribution, the role of PPO in plants has not been thoroughly elucidated. In this study, we report for the absence of PPO in *Cynomorium coccineum*, a holoparasitic plant adapted to withstand unfavorable climatic conditions, growing in Mediterranean countries and amply used in traditional medicine. The lack of PPO has been demonstrated by the absence of enzymatic activity with various substrates, by the lack of immunohistochemical detection of the enzyme, and by the absence of the PPO gene and, consequently, its expression. The results obtained in our work allow us to exclude the presence of the PPO activity (both latent and mature forms of the enzyme), as well as of one or more genes coding for PPO in *C. coccineum*. Finally, we discuss the possible significance of PPO deficiency in parasitic plants adapted to abiotic stress.

## 1. Introduction

Polyphenol oxidases (PPO, EC 1.14.18.1) are type-3 copper monooxygenases found in animals, plants, lichens, and bacteria [1,2,3,4]. These enzymes catalyze the *o*-hydroxylation of monophenols to *o*-catechols (monophenolase or cresolase activity) and the oxidation of the latter to the corresponding *o*-quinones (diphenolase or catechol oxidase activity) [5]. PPOs can also oxidize a variety of other substrates, such as *o*-aminophenols, aromatic diamines, and even peptides bearing tyrosine residues [6].

While the role of PPO has long been recognized in animals, where its catalytic activity is responsible for the biosynthesis of melanin in skin and hair in mammals [7], the role of the enzyme in plants remains uncertain because PPO appears to have more than one function [2,8,9,10]. An interesting role suggested for plant PPOs is the ability to repair damaged cells helping plants defend themselves against some external stress [11]. However, exposure of wheat plants to stress from some heavy metals decreased PPO activity [12].

Plant PPO is usually a constitutive enzyme but can also be induced under certain circumstances. Biotic stresses, such as the rupture of plant tissues (i.e., triggered by pathogens and insects), can cause the enzyme and its natural substrates to come into contact, thus generating browning phenomena typical of many plants. 

The study of the cellular localization of the natural substrates of the PPO contributes only partly to clarify the role of PPO, since the phenolic compounds (main putative substrates of the PPO) are located in different parts of the plant cell, for instance, the vacuoles, guard and epidermal cells, the mesocarp cell wall, and phloem fluids [13,14,15,16,17].

While cellular and subcellular substrate localization can vary, PPO is thought to occur primarily at the luminal side of the thylakoid membrane in the chloroplast cellular compartment [18]. However, several studies have also reported the presence of PPOs in the mitochondria, cell wall, and microsomes [19]. Furthermore, the existence of non-membrane-bound PPOs has also been demonstrated in apple [20] and sugar cane [21].

The study of PPO from holoparasitic plants may be informative about plant PPO function. These plants, lacking chloroplasts, are functionally specialized to acquire at least some essential resources from other plants, the host plant, via specialized organs called haustoria [22].

*Cynomorium coccineum* L. (CC) is an herbaceous and holoparasitic plant, with a very distinctive appearance and color, presenting a high-intensity red-brown inflorescence during the flowering period with a large geographical distribution, including the western Mediterranean, northern Africa, and the Arabian Peninsula [23]. This plant grows in sandy and rocky soils, usually in desert or sub-desert habitats. The stems emerge from the ground in April–May, typically associated with host plants belonging to the Chenopodiaceae, Amaranthaceae, Cistaceae, and some other plant families [24]. The concomitant absence of chloroplasts, the presence of phenolics [25], and the growth in an arid environment make *C. coccineum* a good candidate to investigate any presence and distribution of the PPO enzyme in this plant. We performed this study from multiple perspectives by means of biochemistry, molecular biology, and immunohistochemistry techniques.

## 2. Results

In our experiments in CC extracts, all attempts to detect both PPO activities, monophenolase and diphenolase activity, failed. Enzymatic assays, even those that lasted for a long time (1 h), did not show any significant activity compared to the control without extract. None of the tested compounds (see Materials and Methods section) appeared to be oxidized by the CC homogenate. To test the hypothesis of an activity hidden by the presence of an inhibitor, we dialyzed an aliquot of homogenate (10 mL) for 12 h against 2 L of 50 mM of sodium phosphate buffer (pH 6.5) to remove any small solutes present in the extract and possibly able to function as a PPO inhibitor. However, even in this case, we were unable to measure any PPO activity.

**Figure 1:**
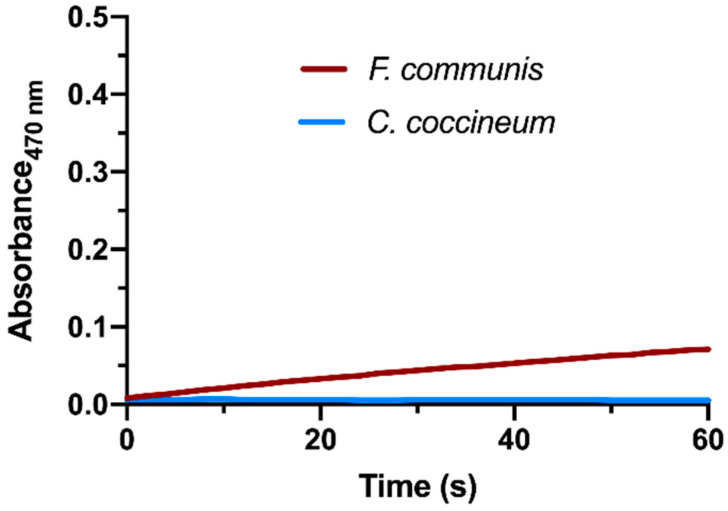
Dopamine oxidation assay of polyphenyl oxidase (PPO): *Cynomorium coccineum* homogenate (blue line) vs. giant fennel (*Ferula communis*) whole plant homogenate used as a positive control.

As an example, Figure 1 shows the result of one of these PPO assays (dopamine oxidation assay) performed with the CC homogenate, in comparison with the giant fennel (*Ferula communis* L.) whole plant homogenate used as a positive control. Giant fennel was chosen because its PPO activity has been previously characterized [26]; it is possible to find this plant in adjacent areas and in the same collection period of CC.

Some PPOs are found in a latent form in vivo, but enzymatic activation can also be reproduced in vitro. One of the most commonly used methods consists of activation by anionic surfactant sodium dodecyl sulfate (SDS) or incubation with trypsin. Both attempts to disclose the presence of a PPO in a latent form in CC were unsuccessful.

To exclude the possibility that failure to observe PPO activity was due to either poor substrate specificity or the low sensitivity of the spectrophotometric method, we used an immunohistochemistry approach to assess the presence of PPO in slices of CC stem using a specific anti—PPO antibody.

**Figure 2:**
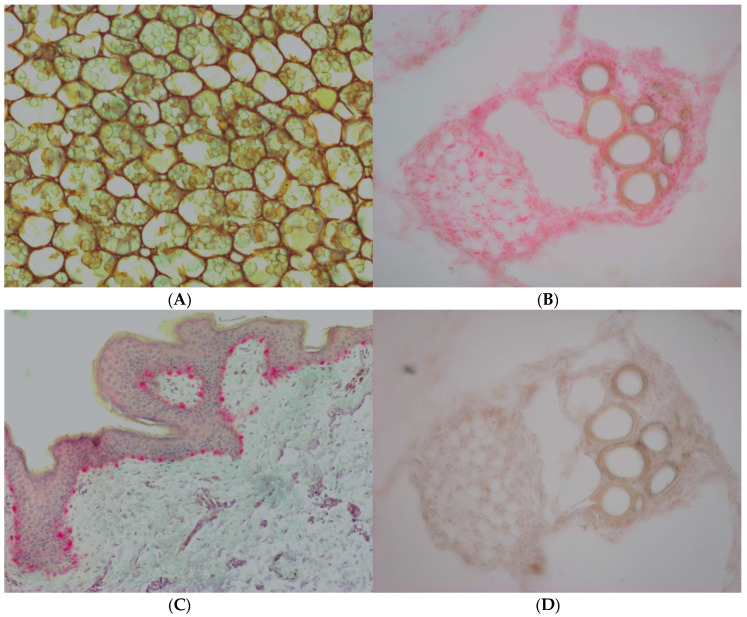
Immunohistochemical detection of PPO in *Cynomorium coccineum*, giant fennel, and human skin. (**A**) *C. coccineum*; (**B**) and (**D**) giant fennel; (**C**) normal human skin. While the immunoreactivity for PPO in *C. coccineum* was absent (**A**), a marked immunostaining, identifiable as a reddish product, was observed in sections of both giant fennel (**B**) and human skin (**C**). In the negative control (**D**), the immunoreactivity was completely abolished. Original magnification: (**A**,**C**): ×100; (**B**,**D**) ×200.

Furthermore, we used samples of normal human skin as a positive control, and giant fennel as a normal control. Both positive and normal controls showed marked immunoreactivity for PPO, while immunostaining in CC slices was absent. Moreover, in the negative control sections of giant fennel, obtained by omission of the primary antibody anti—PPO, the immunoreactivity was completely abolished (Figure 2).

To verify whether the absence of the PPO catalytic activity and the failure of its immunohistochemical detection was linked to the absence of the coding gene or to its lack of expression, we isolated RNA and DNA and searched for the related nucleotide sequence.

PCR experiments conducted on CC mRNA reverse transcribed in cDNA did not show the presence of an expression product related to the PPO gene. In fact, using all primer pairs identified by the CODEHOP program, we did not obtain any PCR products (Supplementary Figure S1). The same primers used to amplify genomic DNA enabled us to obtain PCR products whose sequence showed no degree of homology with the sequences of any PPO genes deposited in the databases. These results allowed us to hypothesize the absence of the gene and, consequently, its expression in *C. coccineum*. Moreover, to exclude the possibility that the absence of PCR products could be due to a lack of cDNA synthesis or the use of degraded DNA, we searched in CC for the polyubiquitin gene whose expression product was detected in this organism. PCR experiments performed on mRNA reverse transcribed into cDNA enabled us to isolate a reaction product whose sequence was highly homologous to those deposited in gene databases.

## 3. Discussion

It is generally assumed that PPO plays a role in defense responses to biotic stresses. This enzyme might act, for example, through the direct toxicity of quinones produced by its catalytic activity or their cross-linking with proteins, thereby forming physical barriers against pathogens and insects. 

Araji et al. (2014) hypothesized an interesting new role for PPO in secondary metabolism, since the silencing of PPO gene expression in the walnut plant caused alterations in the metabolism of some phenolic compounds and their derivatives. PPO-silenced plants displayed an increased level of tyramine (a monophenol substrate of PPO), while its exogenous application elicited cell death in walnut and several other plant species. 

The results obtained in our study lead to excluding the presence of the PPO activity (both latent and mature forms of the enzyme), as well as of one or more genes coding for PPO in *C. coccineum*. 

Indeed, in order to exclude events related to the presence of microRNAs (miRNAs) able to inhibit translation fact, our experiments have not been restricted only to searching the mRNA coding for PPO.

Usually, plant miRNAs are associated with Argonaute protein 1 to promote RNA post-transcriptional gene silencing by coupling target sequences and determining RNA splicing and/or translation inhibition [27,28,29]. Moreover, some authors have shown that both the parasitic and the host plants can bi-directionally exchange their genes [30,31] and large portions of their transcriptome, including miRNA, which can act as a regulator of gene expression [32,33]. These results are in agreement with those reported in a recent study in which the characterization of a large number of mitochondrial and plastid genes, as well as nuclear genes, from the Cynomoriaceae family was conducted [30,31].

However, despite the absence of PPO, the CC specimens found in nature do not seem to be particularly exposed to the attack by insects or pathogens (our personal observation).

Moreover, a total of 29 compounds were recently detected or tentatively identified in *C. coccineum* in mass spectrometry [34]. Interestingly, tyramine was absent from these chemical constituents.

As a result, we can rule out that the absence of PPO makes the CC plant more susceptible to biotic stress, as well as that the enzyme can play a role in the metabolism of tyramine.

The subcellular localization of PPOs is closely connected to the cellular compartment of chloroplasts [19,35], thus leading some authors to hypothesize a connection between PPO and photosynthesis. However, to date, clear evidence either in favor or against such a direct involvement has not been obtained. Despite its chloroplast location, Boeckx et al. [36] showed that the presence of PPO activity in leaves did not correspond with a direct role for the enzyme in the regulation or protection of photosynthesis. Moreover, the detection of potential chloroplast substrates for PPO, namely, coumaroyl hexoside, coumaroyl malate, and caffeoyl malate, both in red clover mutants expressing low leaf-PPO activity and in wild-type plants exhibiting high leaf-PPO activity, suggests a physiological role for this enzyme in undamaged leaves.

Whatever function one wishes to hypothesize for the PPO, one should take into consideration the location of its possible substrates, phenolics, and polyphenols, accumulating predominantly in vacuoles. Different polyphenols have also been identified in the genus *Cynomorium* [37,38], thus suggesting, that their physiological role is not necessary to serve as substrates for PPO.

Therefore, the possibility remains that plant PPO can play more than one physiological role not yet understood.

To our knowledge, this work is the first to report the absence of PPO in a non-photosynthetic plant. Previously, the lack of PPO was suggested only in an *Arabidopsis* mutant, in which a gene encoding a protein with a strong resemblance to laccase was reported [39].

The simultaneous absence of the genes that express both PPO and the protein apparatus necessary for photosynthesis in CC make considerations about the physiological role of PPO in plants even more complex.

Moreover, CC is a plant that is strongly adapted to grow in harsh environmental conditions characterized by arid soil, high salinity, high temperature, and scarcity of water. In many areas of the southern Mediterranean, CC grows close to sub-desert areas [24].

Phenolic compounds are considered to be indicators of drought resistance. For example, in *Larrea divaricata* and *Lycium chilense*, two Patagonian shrubs, phenolic compound production is a strategy used by these species living in extreme environments [40]. Many phenolics, potential PPO substrates, are needed in their reduced state as a form of plant protection from unfavorable environmental conditions. The PPO-mediate oxidation of these substrates might compromise the resistance of the plant.

This finding would be consistent with the observation that PPO levels can also vary in response to various forms of abiotic stress, such as drought. For example, tomato plants in which PPO expression was reduced showed better tolerance to drought than either the untreated plants or those in which PPO was overexpressed [41]. We can therefore speculate that both the high amount of phenolic compounds (e.g., gallic acid and cyanidins) [25], found in the aerial parts of *C. coccineum,* and the concomitant absence of PPO can contribute to greater resistance to abiotic stresses in this plant parasite and, perhaps, in its host plant. However, this hypothesis warrants further research.

Over 4500 angiosperm plants of the 369,000 flowering plants are considered to be parasitic [32]. Many of these plants are holoparasitic and therefore lack the ability to perform photosynthesis, such as *C. coccineum*. However, there are numerous other species, hemiparasites, that contain chlorophyll when mature (hence, these species are photosynthetic) and obtain water and dissolved nutrients by connecting via the haustorium to the host. Such plants may differ in resistance to abiotic stress. Hence, it would be interesting to study the presence of PPO, if any, and its possible location. We hope that our work will be a stimulus for further investigation of these plants to understand possible connections among the function of PPO and abiotic stresses.

## 4. Materials and Methods 

### 4.1. Chemicals

All the chemicals used in this study were purchased from Sigma-Aldrich (Merck Group, Milan, Italy) and were used without further purification. For the purification of genomic DNA and PCR products, specific commercial kits were used as described below.

### 4.2. Plant Material and Homogenate Preparation

*Cynomorium coccineum* specimens (aerial part) were collected in April 2019 from Arborea (southwestern Sardinia, Italy, 39°43′46.2″ N 8°30′48.1″ E) during the flowering period. *Ferula communis* specimens (stem and leaves) were collected in March 2019 from Santa Giusta, Oristano (western Sardinia, Italy, 39°52′01.1″ N 8°36′31.6″ E). After collection, the samples were gently cleaned and frozen within 1 h. Plants were identified using field guides, and identity was confirmed by specialized personnel at Cagliari University.

Reference material for *C. coccineum* (AR-CC/2019/1) and *F. communis* (AR-1/February/2019) was deposited into the collection of the Department of Biomedical Sciences of the University of Cagliari.

### 4.3. Homogenate of C. coccineum

Fresh stems (500 g) from five specimens were cut into small pieces (approximately 2 cm thick) and homogenized in the presence of 0.1 M of cold sodium phosphate buffer (pH 6.5) with Ultraturrax at 10,000 RPM for 1.5 min at 30 s intervals. The slurry was then centrifuged at 14,000× *g* at 4 °C for 20 min. The supernatant was recovered and used for the enzyme assays. Total protein concentration was 4.08 ± 0.08 mg/mL. This procedure was carried out two more times with similar results.

### 4.4. PPO and Protein Assays

The monophenolase activity of PPO was performed with the following monophenol substrates as previously reported: L-tyrosine [42], tyramine [43], umbelliferone [44], and 4-hydroxyanisole [45]. Diphenolase activity measurement was carried out with the following di-phenolic substrates: L-DOPA, dopamine [43], and esculetin [44]. As reported in some PPO assays, 3-methyl-2-benzothiazolinone (MBTH) was used as a coupling reagent to improve the sensitivity of the method. Samples with different amounts of total protein were tested up to a maximum of 2 mg/mL Protein content was measured by Bio-Rad Protein Assay Kits (Clinical Diagnostics, Bio-Rad Laboratories SRL, Milan, Italy) based on Coomassie Brilliant Blue G-250 Dye.

### 4.5. Immunohistochemistry

Samples from five specimens of CC were rapidly frozen at −80 °C and, after 24 h, divided into small pieces, embedded with Tissue-Tek (Sakura Finetek Europe, Alphen aan den Rijn, The Netherlands) and cut into 20 µm sections on a cryostat (Cryostar NX 70, Thermo Fisher Scientific, Waltham, MA, USA). After three consecutive washes in phosphate buffered saline (PBS, pH 7.4), serial sections were subjected to immunohistochemical treatment for the detection of PPO, as previously reported [46]. Following hydration of sections by PBS, samples were incubated with 10% normal horse serum (NHS; Sigma-Aldrich, St. Louis, MO, USA) for 45 min at RT to avoid unspecific binding of the next antibodies. The following procedure steps were performed according to the alkaline phosphatase-streptavidin method. Mouse monoclonal antibody to human PPO (clone T311, 1:100, 1 h at RT; Thermo Fisher Scientific, Waltham, MA, USA) was applied as primary antisera, chosen on the basis of the validated resource identification initiative [47]. After three 5 min rinses in PBS, all of the sections were incubated with the biotinylated horse anti-mouse IgG secondary antibody (1:200, 30 min at RT; Vector Laboratories, Burlingame, CA, USA). PBS washing of the sections (3 × 5 min) preceded the section incubation with the alkaline phosphatase-streptavidin complex (1:1000; Vector Laboratories) for 30 min at RT. Specific immunoreactivity was detected by means of the FastRed substrate-chromogen system (Sigma-Aldrich), allowing the development of the alkaline phosphatase reaction as a reddish product; finally, the sections were mounted in glycerol gelatin (Sigma-Aldrich, Milan, Italy).

To check the specificity of anti-mammalian PPO by Thermo Fisher toward vegetal PPO, cryostat sections (20 µm thick) of stem and leaves from five specimens of giant fennel were used as normal controls and were treated for the immunohistochemical demonstration of PPO enzyme following the same methodological procedure described above.

Moreover, sections of a formalin-fixed and paraffin-embedded normal human skin sample (5 µm) were included in the immunohistochemical analysis and used as a known positive control for PPO. However, before treatment with normal serum, water bath heating-based antigen retrieval was necessary by immersion in 10 mM citrate buffer (pH 6.0) at 95 °C for 30 min followed by gradual cooling for 20 min to recover specific epitopes masked by formalin. Finally, after alkaline phosphatase reaction, the sections were counterstained with Carazzi’s hematoxylin.

Nonspecific binding of secondary antibodies was ruled out by replacing the primary antibodies with normal serum (negative controls). Normal, positive, and negative controls were run simultaneously.

Immunolabeled slides were grabbed using a Zeiss Axioplan2 microscope (Carl Zeiss Vision, Hallbergmoos, Germany) equipped with 10×/0.25 Zeiss Achroplan, 20×/0.45 Zeiss Achroplan, and 40×/0.75 Zeiss Plan-Neofluar objectives that mounted the CCD Lumenera Infinity3-1URC camera (1.4 megapixels; Lumenera Corporation, Ontario, Canada), using the related Infinity Capture 6.3.0 software (Lumenera Corporation). Selected images were slightly adjusted for brightness and contrast, and were combined into panels (Adobe Photoshop 7.0, Adobe Systems Incorporated, CA, USA).

### 4.6. Biomolecular Assay

*Cynomorium coccineum* stems were the starting material for biomolecular experiments on DNA and RNA. The tissue was treated with liquid nitrogen to obtain a thin powder and subsequently frozen and stored at –80 °C until use.

### 4.7. Isolation of RNA from C. coccineum and RT-PCR

Total RNA was extracted from the CC aerial part previously treated with liquid nitrogen using TRI Reagent (Sigma-Aldrich, St. Louis, MO, USA), following the manufacturer’s suggested protocol. The quality of purified RNA was verified by gel electrophoresis using a 1% denaturing agarose gel stained with SYBR Green II (Sigma-Aldrich), and the concentrations were measured using a NanoDrop 2000c UV-VIS Spectrophotometer (Thermo Scientific, Waltham, MA, USA) at 260 nm. To obtain cDNAs, CC RNAs were reverse transcribed with an oligo dT primer using an enhanced avian myeloblastosis virus reverse transcriptase enzyme (Sigma-Aldrich), following the manufacturer’s recommendations.

### 4.8. Genomic DNA Isolation

Genomic DNA was isolated from freeze-dried plant samples using the Plant & Fungi DNA Purification Kit (EURx, Poland) according to the manufacturer’s protocol. The quality and quantity of the DNA were determined spectrophotometrically using a NanoDrop instrument.

### 4.9. Amplification of Cynomorium PPO cDNAs by PCR with Hybrid Primers

**Figure 3:**
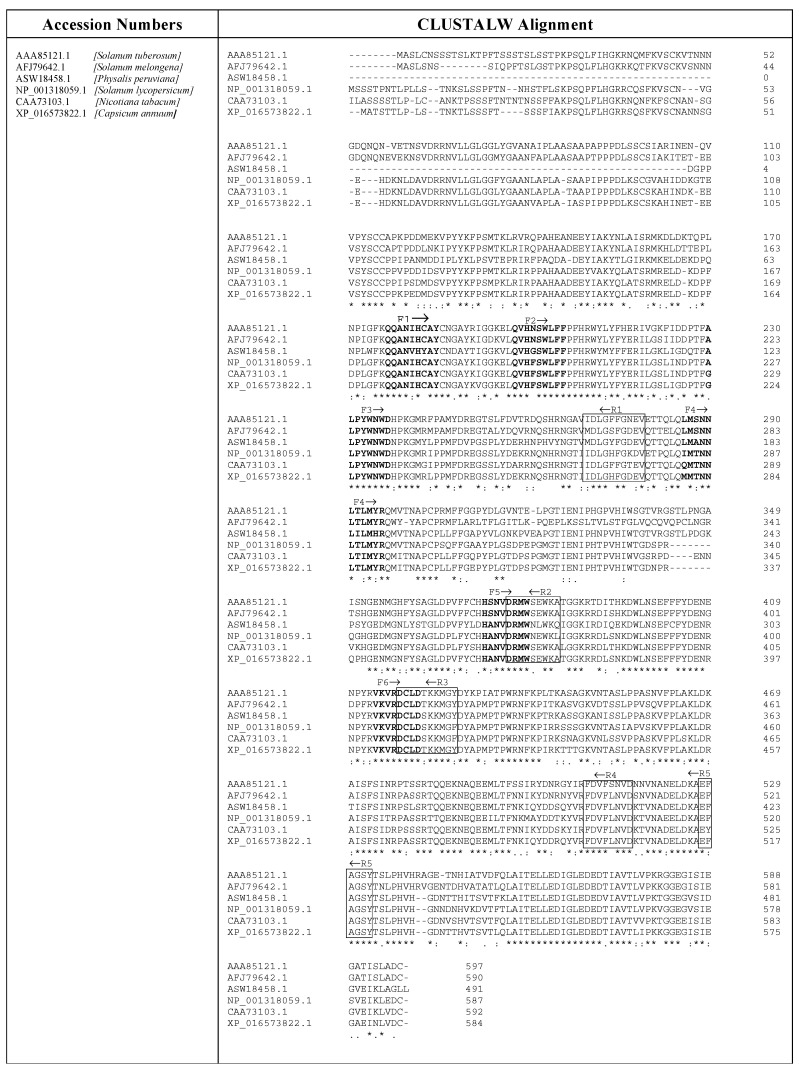
Multiple alignment of PPO amino acids from six different plant sources. The sequences were chosen from the GenBank SwissProt database and aligned using Clustal Omega tool. An asterisk (*) denotes identical residues; double dots (:) represent a conserved residue substitution; a single dot (.) shows partial conservation of the residue. The arrow above the amino acid sequences indicates the position of the sense (F→) and antisense (←R) primers chosen from a group of candidate primers obtained from the CODEHOP program. The sequences of the sense and antisense primers were obtained from the Clustal Omega alignments shown in bold and inside the boxes, respectively.

A degenerate hybrid oligonucleotide primer (CODEHOP) strategy [48] was used to detect unknown nucleotide sequences of the CC genes. The CODEHOP strategy has proven to be useful for the exploration of large gene families in plants [49] and for the isolation of different plant genes related to antioxidant activity [50,51,52,53]. According to the CODEHOP strategy, we selected six PPO amino acidic sequences of different plant sources from the GenBank SwissProt database. The sequences were aligned using Clustal Omega (https://www.ebi.ac.uk/Tools/msa/clustalo/) and then cut into blocks using Block Marker software (http://blocks.fhcrc.org/blocks/). Primers were designed using the default parameters of the CODEHOP program (http://blocks.fhcrc.org/codehop.html). Amplification primers used for PPO cDNA from CC were chosen from a group of candidate primers suggested by the CODEHOP program. The position and orientation of the primers with respect to the gene translated in silico into amino acid sequences are shown in Figure 3. Each primer sequence contains a consensus clamp, given in the upper case, and a degenerate core, written in the lower case, with y = [C,T], r = [A,G], and n = [A,G,C,T]. PCR was performed in a solution containing 1.5 mM MgCl_2_, 100 mM Tris-HCl (pH 8.3), 50 mM KCl, 200 mM dNTP mix, 1 mM sense primer, 1 mM antisense primer, 1 μg of *Cynomorium* cDNA, and 1–3 units of Jump Start AccuTaq LA DNA polymerase mix (Sigma-Aldrich). Thermal cycling was carried out in a Personal Eppendorf Mastercycler (Eppendorf, Hamburg, Germany) under varying conditions. All PCRs were prepared using pairs of primers available in all possible combinations (see Table 1). The electrophoretic separation of the PCR products was carried out not only on agarose gel 2% but also on 6% polyacrylamide gel (acrylamide/bis ratio 29:1), which allows a better separation of small PCR fragments (80–800 bp) compared to 2–3% agarose normally used, as previously reported [54].

### 4.10. Amplification of Cynomorium polyubiquitin cDNA and Genomic DNA by PCR with Hybrid Primers

To evaluate the efficiency of reverse transcription polymerase chain reaction and the presence of full-length CC cDNAs, a PCR reaction mixture was prepared with the aim of isolating a gene fragment coding for polyubiquitin. This gene was chosen because its nucleotide sequence in CC was partially characterized in our laboratory (accession number KX611140). The forward (5′-AAGCAGCTTGAGGACGGGAGAACACTA-3′) and reverse (5′-GGTCAGGGTCTTCACGAAGATCTGCAT-3′) primers for amplifying the gene fragment coding for the CC polyubiquitin were designed to amplify the entire 570-bp nucleotide sequence deposited in the NCBI database (Accession Number KX611140.1). The results of the electrophoretic separation of PCR products are shown in Supplementary Materials (Supplementary Figure S1). The combinations of sense and antisense primers used in PCR experiments and the size of the expected fragments are shown in Supplementary Table S1. The only PCR fragment obtained relating to polyubiquitin was purified with a Charge Switch PCR Clean-Up Kit (Invitrogen, Carlsbad, CA, USA) and sent to BMR Genomics (Padova, Italy) for sequencing. The results are given in Supplementary Figure S2.

Nucleotide and deduced amino acid sequence analyses were performed with the OMIGA v.2.0 software (Oxford Molecular Company, Madison, WI, USA). Translation of nucleotide sequences was performed using OMIGA or the ExPASy translate routine software tool. Similarities were analyzed with the advanced BLAST algorithm, available at the National Center for Biotechnology Information website and with the FASTA algorithm v. 3.0 from the European Bioinformatics Institute website (EMBL-EBI). Sequences were aligned using Clustal Omega.

## Figures and Tables

**Table 1:** Oligonucleotides used in the CODEHOP experiments.

CODEHOP Sequences	Comments
5′-CAGCAGGCCAACATCcaytgygcnta-3′	F1 sense primer designed on the conserved peptide QQANIHCAY
5′-GCAGGTGCACAACTCCtggytnttytt-3′	F2 sense primer designed on the conserved peptide QVHNSWLFF
5′-CGCCATGCCCTACtggaaytggga	F3 sense primer designed on the conserved peptide ALPYWNWD
5′-GCAGATGAACAACAACCTGACAhtnatgtaymg-3′	F4 sense primer designed on the conserved peptide QMNNNLTIMYR
5′-ACCACGCCAACGTGgaymgnatgtg-3′	F5 sense primer designed on the conserved peptide HSNVDRMW
5′-GGGTGAAGGTGCGGgaytgyytnga-3′	F6 sense primer designed on the conserved peptide VKVRDCLD
5′-CACCTCGGTGCCGAAGTAGycnarrtcnat-3′	R1 antisense primer designed on the conserved peptide IDLGYFGTEV
5′-GCCTTCCACTCGTTCcacatnckrtc-3′	R2 antisense primer designed on the conserved peptide DRMWSEWKA
5′-CGTAGCCCATCTTCTTGGTGtcnarrcartc-3′	R3 antisense primer designed on the conserved peptide DCLDSKKMGYD
5′-CGTTCGACGTTCACGwanacrtcraa-3′	R4 antisense primer designed on the conserved peptide FDVFLNVDV
5′-GTAGGAGccngcrwaytc-3′	R5 antisense primer designed on the conserved peptide EFAGSY

## Supplementary Materials

The following are available online at https://www.mdpi.com/2223-7747/9/8/964/s1: Figure S1: Electrophoretic separation on 6% polyacrylamide gel of the PCR product obtained for the polyubiquitin gene (line 1); Table S1: Combination of primers sense and antisense used in PCR reactions and size of the expected fragments given in base pairs (bp); Figure S2: Polyubiquitin sequencing results.
