# Supplementary material for: Absence of Polyphenol Oxidase in Cynomorium coccineum, a Widespread Holoparasitic Plant
Source: Plants (Basel). 2020 Jul 30;9(8):964. doi: 10.3390/plants9080964 (PMC7570208; doi:10.3390/plants9080964)
Supplement: Supplementary file 1 [file plants-09-00964-s001.docx]

Supplementary Materials


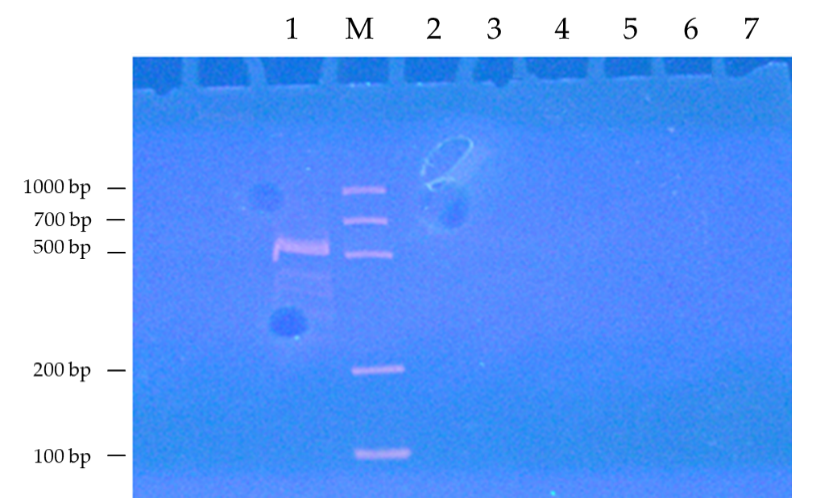


**Figure 1.** Electrophoretic separation on 6% polyacrylamide gel of the PCR product obtained for the polyubiquitin gene (line 1). The empty lanes are relative to the PCR results for the PPO gene (lines 2-7). The DNA fragment size marker (M) was provided by Bio-Rad (EZ Load ™ Precision Molecular Mass Ruler 1708356).

**Table 1.** Combination of primers sense and antisense used in PCR reactions and size of the expected fragments given in base pairs (bp). In the case of PPO, the size of the expected fragments was calculated approximately on the basis of the amino acid alignment of the different proteins taken into consideration for the design of the CODEHOP primers.

| **POLYUBIQUITIN SPECIFIC PRIMERS** | **expected size (bp) polyubiquitin fragment** |
| --- | --- |
| Ubi F 5’-AAGCAGCTTGAGGACGGGAGAACACTA-3’  Ubi R 5’- GGTCAGGGTCTTCACGAAGATCTGCAT-3’ | 570 bp |
| **PPO CODEHOP Primer** | **expected size (bp)**  **PPO fragments** |
| F1 5’-CAGCAGGCCAACATCcaytgygcnta-3’  R1 5’-CACCTCGGTGCCGAAGTAGycnarrtcnat-3’ | 2 ≅ 330 bp |
| F2 5’-GCAGGTGCACAACTCCtggytnttytt-3’  R2 5’-GCCTTCCACTCGTTCcacatnckrtc-3’ | 3 ≅ 570 bp |
| F3 5’-CGCCATGCCCTACtggaaytggga  R3 5’-CGTAGCCCATCTTCTTGGTGtcnarrcartc-3’ | 4 ≅ 590 bp |
| F4 5’-GCAGATGAACAACAACCTGACAhtnatgtaymg-3’  R2 5’-GCCTTCCACTCGTTCcacatnckrtc-3’ | 5 ≅ 300 bp |
| F5 5’-ACCACGCCAACGTGgaymgnatgtg-3’  R4 5’-CGTTCGACGTTCACGwanacrtcraa-3’ | 6 ≅ 440 bp |
| F6 5’-GGGTGAAGGTGCGGgaytgyytnga-3’  R5 5’-GTAGGAGccngcrwaytc-3’ | 7 ≅ 360 bp |

**Figure 2.** Polyubiquitin sequencing results. The sequencing reaction was carried out by Macrogen (Macrogen Europe B.V. Meibergdreef 31 1105 AZ, Amsterdam, the Netherlands). The sequence was deposited in the NCBI database (GenBank: KX611140.1).
